# Hay Fever

**Published:** 1889-12-28

**Authors:** 


### HAY FEVER.

At a meeting of the New York Academy of Medicine, held in November last, Dr. Seth Bishop, of Chicago, proposed the appointment of a committee to co-operate with the United States Hay Fever Association, formed for the investigation and treatment of hay fever. We certainly thought London and England generally could boast of more and varied societies and associations than any other locality in the world. And we have sometimes thought that only one more society was required—viz., the "Anti-Humbug Society," whose business would be to take cognisance of the doings of other societies. But we now begin to think prominence in the matter of associations must be yielded to New York, or rather the United States. We do not, however, mean to insinuate that there is not room for a "Hay Fever Association," for it seems that affections of the nasal mucous membrane are much more prevalent in America than in this country—at least, affections which come under the heading hay fever. Such maladies, however, are sufficiently common in the United Kingdom to render the subject one of great interest, although, perhaps, not to demand the institution of a special society for the purpose of investigating the complaint. As with asthma, so with its kindred affection, hay fever, the malady is very erratic in its nature. It has almost as many names as, say, for instance, remittent fever. Thus, it has been called "pollen fever," "rose cold," "grape cold," "peach cold," "Roman wormwood cold,' "spasmodic sneezing," "spasmodic catarrh," and "vaso-motor coryza." In this country it is most prevalent when the hay is ripening, but, as the names given above indicate, it is prevalent in other localities when other vegetable life is blooming. The general idea is that it depends on pollen or special vegetable atoms floating in the atmosphere, which prove irritating to the nasal mucous membrane. We believe, however, that it depends on idiosyncrasy, or peculiar irritability of the nasal mucous membrane arising from idiosyncrasy. For we have seen spasmodic sneezing quite as annoying as anything of the kind ever arising from hay pollen excited by exposure to heat and fine sand or dust, recurring whenever such conditions dominated the atmosphere. We shall look with some curiosity to the report of the "United States Hay Fever Association."

